# Putative proteomic signatures associated with post-thaw semen quality in Peranakan Etawah and Saanen goats: an exploratory study

**DOI:** 10.5713/ab.260268

**Published:** 2026-07-27

**Authors:** Muhammad Ihsan Andi Dagong, Muhammad Yusuf, Athhar Manabi Diansyah, Procula Rudlof Matitaputty, Paskah Partogi Agung, Indra Wahyudi Syarif, Muhammad Fajar Amrullah

**Affiliations:** 1 Faculty of Animal Science, Hasanuddin University, Makassar, Indonesia; 2 Research Center for Animal Husbandry, Research Organization for Agriculture and Food, Bogor, Indonesia; 3 Research Center for Applied Zoology, National Research and Innovation Agency, Bogor, Indonesia; 4 Graduate Student of Animal Production and Technology Science, Graduate School, IPB University, Bogor, Indonesia; 5 Graduate Student of Animal Biomedical Science Study Program, School of Veterinary Medicine and Biomedical Science, IPB University, Bogor, Indonesia

**Keywords:** Peranakan Etawah, Proteomics, Saanen, Semen Quality, Sperm Kinematics

## Abstract

**Objective:**

The present study aimed to investigate post-thaw semen quality and sperm kinematics between Peranakan Etawah (PE) and Saanen goats, and to identify breed-associated proteomic markers related to semen cryotolerance.

**Methods:**

Frozen semen straws were obtained from BIB Singosari, Indonesia, and grouped by breed. Post-thaw evaluations included cellular viability, morphology/abnormality, plasma membrane integrity (HOST), and computer-assisted sperm analysis for motility and kinematic parameters. Sperm proteomes were profiled via UHPLC–Orbitrap LC–MS/MS. Data were processed in Proteome Discoverer and analyzed using multivariate models (principal component analysis [PCA], partial least squares–discriminant analysis [PLS-DA]), differential abundance testing (q-values), functional enrichment (g:Profiler), biomarker selection (variable importance in projection>1; area under the curve≥0.70), and correlation with semen traits (MetaboAnalyst).

**Results:**

PE and Saanen differed significantly in survival-related traits, including viability, abnormalities, and plasma membrane integrity (p<0.05), while total motility and other kinematic parameters were similar between breeds (p>0.05). Proteomic patterns showed partial separation by PCA and more distinct differentiation through verified PLS-DA. Multiple proteins exhibited variable abundance between the breeds (q<0.05) and clustered reliably by breed. Enrichment analysis highlighted mitochondrion-associated functions, particularly fatty acid β-oxidation. Identified discriminative proteins included ACADVL, ETFB, GPD2, CYLC1, and LOC102188264, which were significantly associated with semen quality traits within breeds.

**Conclusion:**

Differences across breeds in frozen–thawed goat semen were mostly evident in post-thaw viability traits and in proteomic signatures predominantly influenced by mitochondrial energy metabolism. Integrating proteomics with conventional semen assessment may elucidate semen cryotolerance and support biomarker identification.

## INTRODUCTION

Artificial insemination (AI) has become a central component of genetic improvement programs in small ruminants, enabling rapid dissemination of superior germplasm across regions and production systems [1]. In Indonesia, Peranakan Etawah (PE) and Saanen goats are among the most widely utilized dairy and dual-purpose breeds. PE goats represent a locally adapted composite breed with strong environmental resilience, whereas Saanen goats are internationally recognized for their high milk production potential [2,3]. Although both breeds are extensively used in breeding programs, differences in genetic background, physiological adaptation, and cellular characteristics may influence semen quality and cryotolerance.

Cryopreservation is essential for AI programs; however, the freezing and thawing process induces structural and metabolic stress on spermatozoa [4]. Cryo-induced damage commonly affects plasma membrane integrity, mitochondrial function, oxidative balance, and DNA stability, ultimately reducing post-thaw viability and fertilization capacity [5]. Conventional semen evaluation parameters—such as motility, viability, morphology, and membrane integrity—are routinely used to assess post-thaw quality. While these parameters provide valuable functional information, they represent downstream phenotypic outcomes and may not fully capture underlying molecular mechanisms influencing sperm resilience [6].

Computer-assisted sperm analysis (CASA) enables objective measurement of sperm kinematic characteristics, including velocity and trajectory-related parameters, offering a more detailed assessment of motion dynamics compared to subjective evaluation [7]. Nevertheless, sperm motility alone does not necessarily reflect metabolic competence, mitochondrial efficiency, or oxidative stress status [8]. Spermatozoa that appear motile may still exhibit subcellular dysfunction that compromises fertilization potential. Therefore, integrating molecular-level analysis with conventional semen evaluation may provide deeper insight into breed-associated variation in cryotolerance.

Proteomics offers a systems-level approach for investigating the protein composition of spermatozoa and seminal plasma, reflecting functional pathways involved in energy metabolism, structural organization, oxidative regulation, and fertilization processes [9]. Because proteins mediate cellular structure and biochemical activity, proteomic profiling captures the functional expression of genetic background. In reproductive biology, proteomic approaches have been used to identify protein signatures associated with sperm quality, fertility outcomes, and cryoresistance across livestock species [10]. Studies have reported that mitochondrial proteins, enzymes involved in fatty acid β-oxidation, and cytoskeletal proteins are frequently associated with post-thaw sperm performance and fertilizing capacity [11–13].

Despite the importance of PE and Saanen goats in Indonesian breeding systems, comparative proteomic studies evaluating breed-associated molecular differences in frozen–thawed semen remain limited. Current semen selection strategies largely rely on conventional quality parameters, which may not fully reflect intrinsic bioenergetic organization or structural resilience at the molecular level. Understanding the proteomic configuration underlying semen performance could enhance objective evaluation frameworks and support more informed breeding decisions.

Therefore, this study aimed to compare post-thaw semen quality and sperm kinematic characteristics between PE and Saanen goats and to integrate these phenotypic traits with proteomic profiling. By identifying differentially abundant proteins and candidate molecular biomarkers associated with semen performance, this research sought to provide preliminary insight into molecular mechanisms potentially associated with variation in sperm cryotolerance between breeds. We hypothesized that frozen–thawed semen from PE and Saanen goats would exhibit differences in semen quality parameters and sperm proteomic profiles, particularly in proteins related to mitochondrial metabolism and energy production.

## MATERIALS AND METHODS

### Experimental design and ethical approval

This study compared frozen–thawed semen quality and sperm kinematic characteristics between PE and Saanen goats and integrated these parameters with proteomic profiling. Frozen semen straws were obtained from the Artificial Insemination Center (BIB) Singosari, Indonesia, and allocated into two groups based on breed: PE (n = 5) and Saanen (n = 5). Post-thaw semen quality evaluation and sperm kinematic analysis using a CASA system were performed at the Integrated Zoology Laboratory, National Research and Innovation Agency (BRIN), Cibinong, Indonesia. Proteomic analysis was conducted at the Corpora Science Laboratory to characterize and compare protein expression profiles between breeds.

### Post-thaw semen quality evaluation

Frozen semen straws were thawed in a water bath at 37°C for 30 s and gently homogenized prior to evaluation. Sperm motility and kinematic characteristics were assessed using the SpermVision CASA system (Minitube) connected to a phase-contrast microscope (Carl Zeiss) equipped with a thermostatically controlled stage maintained at 38°C. A 5–10 μL aliquot of semen was loaded onto a pre-warmed glass slide, covered with a coverslip, and analyzed immediately. For each sample, at least 200 spermatozoa were evaluated across five randomly selected microscopic fields [14].

Sperm concentration was determined using a photometer (SDM 6; Minitube). Semen was diluted in 0.9% NaCl solution and measured according to the manufacturer’s protocol. Sperm viability and morphology were evaluated using eosin staining. Briefly, 10 μL of semen was mixed with 2% eosin, smeared onto a slide, and examined under a Carl Zeiss Microimaging at 400× magnification. Spermatozoa showing red-stained heads were classified as non-viable, and morphological abnormalities were recorded from at least 200 spermatozoa per sample [15].

Intact membrane was assessed using the hypo-osmotic swelling test (HOST) by incubating semen in a hypo-osmotic solution (0.179 g NaCl/100 mL distilled water) at 37°C for 30 min. Spermatozoa displaying coiled tails were considered to have intact plasma membranes [15].

### Proteomic analysis

Proteomic profiling was performed following the procedure described by Rimadhani et al. [16] using a Thermo Fisher Scientific Vanquish Horizon UHPLC system coupled to an Orbitrap Exploris 240 high-resolution mass spectrometer. For protein preparation, 200 μL of each semen sample was precipitated with five volumes of cold acetone (−20°C) and incubated overnight. After centrifugation (15,000×g, 4°C, 15 min), the supernatant was discarded and the pellet was air-dried. The pellet was resuspended and sonicated (40 kHz, three cycles, ice bath), followed by centrifugation to collect the protein-containing supernatant. Proteins were reduced with 10 mM dithiothreitol (DTT) at 56°C for 1 h and alkylated with 55 mM iodoacetamide (IAA) in the dark at room temperature for 1 h. The solution was diluted with 50 mM ammonium bicarbonate and digested overnight at 37°C using trypsin. Digestion was terminated by acidification with trifluoroacetic acid (TFA), and the peptide mixture was clarified by centrifugation and filtered through a 0.2 μm PVDF membrane prior to LC–MS/MS analysis. Peptide separation was achieved on an Acclaim PepMap 100 C18 column (150 mm×1.0 mm, 3 μm) maintained at 30°C, using water (0.1% formic acid) as mobile phase A and acetonitrile (0.1% formic acid) as mobile phase B at a flow rate of 75 μL/min. A linear gradient elution was applied from 5% to 50% B over 30 min, followed by an increase to 90% B and re-equilibration to initial conditions within a total run time of 45 min. Mass spectrometric analysis was conducted in positive ion mode using a data-dependent acquisition (Full MS/dd-MS2) method. Full MS scans were acquired at a resolution of 120,000 FWHM over an m/z range of 350–1,200, with a maximum injection time of 100 ms and mass tolerance of 5 ppm. MS/MS spectra were collected at 15,000 FWHM using nitrogen as the collision gas and a normalized collision energy of 30. Ionization was performed using a heated electrospray ionization (H-ESI) source operated at 4,000 V with an ion transfer tube temperature of 300°C.

### Bioinformatic and statistical analysis

Raw LC–MS/MS spectra were processed using Proteome Discoverer (Thermo Fisher Scientific) and searched against the UniProt *Capra hircus* reference proteome. High-confidence protein identifications were retained based on the presence of at least two unique peptides and a protein-level false discovery rate (FDR)<1% [16]. Protein abundance data were log2-transformed, and missing values and normalization were processed using the corresponding preprocessing functions implemented in MetaboAnalyst 6.0 prior to downstream analyses.

Statistical analysis and visualization of proteomic data were performed using MetaboAnalyst 6.0. Differential protein abundance between PE and Saanen groups was evaluated using log2 fold change and corresponding p-values, with multiple-testing correction performed the Benjamini–Hochberg procedure. Proteins with adjusted q-values<0.05 were considered significantly differentially abundant. Global expression patterns were explored using principal component analysis (PCA), hierarchical clustering heatmaps, volcano plots, and Venn diagrams. Group discrimination was further assessed by partial least squares–discriminant analysis (PLS-DA), and variable importance in projection (VIP) scores were used to estimate the contribution of individual proteins to group separation, with VIP scores>1 considered important variables. Model performance was evaluated using R^2^ and Q^2^ values generated in MetaboAnalyst. Receiver operating characteristic (ROC) analysis was performed using the biomarker analysis module in MetaboAnalyst, and classification performance based on the area under the curve (AUC).

Functional enrichment analysis was conducted using g:Profiler to identify significantly overrepresented Gene Ontology (GO) terms and biological pathways associated with differentially abundant proteins. Enriched categories were interpreted based on adjusted significance values and leading proteins within each term.

For conventional semen quality parameters and CASA-derived kinematic variables, statistical analyses were performed using IBM SPSS Statistics ver. 26 (IBM). Data were assessed for normality prior to comparison. Normally distributed variables were analyzed using an independent samples t-test, whereas non-normally distributed variables were analyzed using the Mann–Whitney U test. Results were presented as mean±standard deviation (SD), and statistical significance was set at p<0.05.

## RESULTS

### Post-thaw semen quality and sperm kinematics

Post-thaw semen quality parameters are presented in Table 1. Significant differences between PE and Saanen goats were observed in sperm viability, abnormality, and plasma membrane integrity (p<0.05). In contrast, total motility did not differ significantly between breeds (p>0.05). Sperm kinematic parameters are summarized in Table 2. No significant differences were detected between breeds for progressive motility, local motility, immotile percentage, motion distance parameters (distance curved line [DCL], distance average path [DAP], distance straight line [DSL]), velocity parameters (velocity curved line [VCL], velocity average path [VAP], velocity straight line [VSL]), linearity indices (linearity [LIN], straightness [STR], wobble [WOB]), beat cross frequency (BCF), or amplitude of lateral head displacement (ALH) (p>0.05).

### Overview of semen proteomic profiling

Multivariate analysis was performed to evaluate global protein expression patterns between PE and Saanen frozen–thawed semen samples (Figure 1). PCA demonstrated partial separation between groups, with some overlap observed among biological replicates (Figure 1A). Supervised analysis using PLS-DA showed clearer discrimination between PE and Saanen samples (Figure 1B). Pairwise score plots from the PLS-DA model demonstrated partial separation between PE and Saanen groups, indicating potential discriminatory patterns in the proteomic dataset (Figure 1C).

Cross-validation was subsequently performed to assess the performance of the PLS-DA model, and the results are presented in Table 3. The model showed high R^2^ values, ranging from 0.90389 to 1.00000 across one to five components, indicating strong goodness of fit to the training data. However, Q^2^ values were negative across all components, ranging from −0.55699 to −0.42230, with an accuracy value of 0.40. These findings indicate limited predictive performance of the PLS-DA model. Therefore, PLS-DA was used only as an exploratory tool to support candidate biomarker screening, whereas final candidate biomarker selection relied on integrated evidence from VIP score, differential abundance, biological relevance, pathway involvement, and AUC analysis.

### Differential protein abundance and expression patterns

Comparative analysis revealed a subset of proteins that were significantly differentially abundant between PE and Saanen frozen–thawed semen samples (q<0.05). The volcano plot illustrates the distribution of proteins according to log2 fold change and statistical significance, highlighting proteins that were upregulated and downregulated between groups (Figure 2A). Several proteins showed marked differences in abundance, as indicated by their separation beyond the significance thresholds.

Hierarchical clustering analysis of differentially abundant proteins demonstrated distinct expression patterns between PE and Saanen samples (Figure 2B). Because the heatmap was generated using statistically filtered differentially abundant proteins, biological replicates showed a tendency to cluster according to breed. The heatmap further showed clear differences in relative protein abundance across samples.

Multivariate feature selection using PLS-DA identified proteins with VIP scores>1 as major contributors to group discrimination (Figure 3). These proteins were considered key variables distinguishing PE and Saanen semen proteomes and were retained for subsequent functional analysis. The highest VIP scores were observed for MINPP1, ATP5F1D, ACADVL, TRIM38, and LOC102188264, indicating that these proteins contributed strongly to the discrimination between PE and Saanen groups. Several proteins exhibited contrasting abundance patterns between groups, as shown in the accompanying heatmap.

### Functional enrichment analysis

Functional enrichment analysis using g:Profiler identified several significantly overrepresented categories among the differentially abundant proteins (adjusted p<0.05). The enriched terms included biological processes related to fatty acid β-oxidation, represented by ACADVL and ETFB (GO:0033539; padj = 4.619×10^−2^). At the cellular component level, significant enrichment was observed for mitochondrion-associated proteins, including ACADVL, LOC102188264, ETFB, GPD2, and ATP5IF1 (GO:0005739; padj = 6.611×10^−3^). In addition, the acrosomal matrix term was significantly enriched and represented by CYLC1 (GO:0043159; padj = 3.090×10^−4^). Figure 4 presents the distribution of enriched functional categories across GO molecular function (GO:MF), biological process (GO:BP), cellular component (GO:CC), KEGG pathways, and Human Phenotype (HP) databases. Each circle represents an enriched term, with the vertical axis indicating enrichment significance based on −log10 adjusted p-values. Each circle represents an enriched term; numbered circles correspond to labeled enriched terms in the output. These enrichment results are summarized in Figure 4 and Table 4.

### Classification performance

Proteins with VIP scores>1 and discriminative performance of AUC≥0.70 were selected as candidate biomarkers distinguishing PE and Saanen semen samples (Table 5). Five proteins met these criteria, including ACADVL, ETFB, CYLC1, GPD2, and LOC102188264. Among these, ACADVL showed the highest discriminative performance (AUC = 0.92), followed by LOC102188264 (AUC = 0.80). The relative abundance patterns of candidate biomarker proteins are shown in Figure 5. ACADVL, ETFB, and GPD2 displayed higher expression levels in Saanen samples, whereas CYLC1 and LOC102188264 exhibited higher abundance in PE samples. The distribution patterns observed in the box plots were consistent with the group discrimination identified through multivariate and ROC analyses.

### Correlation between semen parameters and candidate biomarkers

Spearman correlation analysis identified several significant associations between candidate biomarker proteins and post-thaw semen parameters (p<0.05) (Figure 6). In PE goats, CYLC1 showed a significant negative correlation with abnormality and significant positive correlations with motility-related parameters. LOC102188264 was significantly positively correlated with intact membrane, progressive motility, and selected velocity parameters, while exhibiting a significant negative correlation with local motility. In Saanen goats, ACADVL demonstrated significant positive correlations with viability and selected velocity parameters, along with a significant negative correlation with local motility. ETFB and GPD2 showed significant positive correlations with viability and linearity-related parameters, whereas significant negative correlations were observed with ALH.

## DISCUSSION

The present study demonstrates that breed-associated differences in frozen–thawed semen quality are accompanied by distinct proteomic signatures, particularly within mitochondrial and lipid metabolic pathways. While most sperm kinematic parameters were comparable between PE and Saanen goats, significant differences in viability and plasma membrane integrity suggest that cryosurvival and membrane resilience represent key phenotypic traits differentiating the two breeds. Conventional motility parameters do not always capture subcellular metabolic disturbances induced by cryopreservation; therefore, the proteomic differentiation observed in this study likely reflects intrinsic molecular organization underlying post-thaw sperm function [17,18].

Mitochondrial function is central to sperm survival and post-thaw recovery. Beyond ATP production for motility, mitochondria regulate oxidative phosphorylation efficiency, reactive oxygen species (ROS) generation, and redox buffering, all of which are strongly affected during freezing and thawing [19,20]. Cryopreservation is widely reported to disrupt mitochondrial membrane potential, impair electron transport, and increase oxidative damage, leading to reduced viability and compromised membrane stability [21,22]. The enrichment of mitochondrion-associated proteins and fatty acid β-oxidation pathways in the present study therefore supports the interpretation that bioenergetic configuration is a major molecular axis distinguishing PE and Saanen semen.

This interpretation is strengthened by the candidate biomarker panel identified through VIP and ROC analyses. ACADVL, ETFB, GPD2, and LOC102188264 are all functionally linked to mitochondrial metabolism and energy transfer. ACADVL catalyzes the first step of mitochondrial β-oxidation for very-long-chain fatty acids, a pathway that contributes reducing equivalents to sustain electron transport and ATP generation [23,24]. ETFB plays an essential role in electron transfer from flavoprotein dehydrogenases to the respiratory chain, supporting oxidative metabolism and mitochondrial efficiency [25,26]. GPD2 contributes to the glycerol phosphate shuttle, linking cytosolic redox state to mitochondrial respiration and potentially supporting ATP production under stress conditions [27]. LOC102188264, annotated as a mitochondrial cytochrome complex component, further reinforces the dominance of mitochondrial respiratory processes in differentiating the two breeds.

Taken together, these proteins suggest that mitochondrial substrate utilization and respiratory chain activity may represent key determinants of semen cryotolerance. This aligns with broader evidence that spermatozoa with preserved mitochondrial function exhibit higher survival rates, improved membrane integrity, and greater post-thaw fertilization potential [28,29]. The identification of a mitochondrial-centered biomarker set is therefore biologically coherent with the phenotypic differences observed in viability and membrane integrity.

Functional enrichment analysis also supports this interpretation. The enrichment of fatty acid β-oxidation terms indicates that lipid-derived energy metabolism may be particularly relevant in the context of frozen semen. Sperm membranes contain high proportions of polyunsaturated fatty acids, making them susceptible to lipid peroxidation during cryopreservation [30]. At the same time, fatty acid oxidation provides a metabolic route for sustaining mitochondrial respiration. Variation in proteins involved in these pathways may therefore reflect breed-associated differences in the balance between lipid utilization and oxidative vulnerability. This dual role of lipid metabolism—supporting energy production while also being linked to oxidative sensitivity—may be especially important for explaining differences in post-thaw membrane integrity.

Although the mitochondrial signature dominated the proteomic differentiation, the identification of CYLC1 introduces an additional structural dimension. Cylicins are cytoskeletal proteins associated with sperm head architecture and acrosomal-associated structures [31]. Structural stability is relevant during cryopreservation because osmotic stress and membrane phase transitions can disrupt head morphology and membrane anchoring [32]. In the present study, CYLC1 met biomarker selection criteria and was significantly associated with semen phenotypes, suggesting that structural protein composition may contribute to breed-associated variation in post-thaw quality. However, CYLC1 likely represents one component within a broader systems-level profile, rather than a single dominant determinant.

Interestingly, despite clear proteomic divergence and differences in membrane-related quality traits, kinematic parameters were largely comparable between breeds. This pattern may indicate that sperm motion characteristics among the surviving post-thaw population remain relatively stable, while breed differences are expressed primarily in survival-related outcomes such as viability and membrane integrity. This is consistent with the concept that motility is a downstream functional trait that can remain preserved in the subset of spermatozoa that survive cryopreservation, even when overall cellular damage differs [33]. It also highlights the value of proteomic profiling for detecting molecular differences that are not readily observable through kinematic measurements alone.

Correlation analysis further linked candidate proteins to semen phenotypes, supporting their functional relevance. In PE samples, CYLC1 and LOC102188264 were significantly associated with membrane integrity and motility-related parameters, while in Saanen samples ACADVL showed significant associations with viability and velocity-related traits. ETFB and GPD2 were significantly correlated with viability and selected kinematic indices. These associations provide an integrative connection between proteomic signatures and semen quality traits and suggest that mitochondrial and structural proteins may contribute to post-thaw performance through complementary mechanisms [34,35].

From an applied perspective, the identification of a candidate biomarker panel provides potential utility for semen quality evaluation and cryopreservation improvement. Current semen assessment relies heavily on motility and morphology, which may not fully represent molecular-level resilience to cryostress [36,37]. Proteins involved in mitochondrial metabolism (ACADVL, ETFB, GPD2, LOC102188264) and sperm structural stability (CYLC1) may serve as candidate markers associated with differences in semen cryotolerance between breeds. These markers could also support future efforts to optimize extenders and cryoprotective strategies aimed at preserving mitochondrial function and reducing oxidative damage [38]. However, these potential applications remain speculative and require further functional and fertility-based validation before practical implementation.

Several limitations should be considered. The sample size was limited (n = 5 per group), which may reduce statistical power and increase the risk of overfitting in supervised models [39]. Therefore, the present findings should be interpreted as exploratory. Furthermore, correlation analyses were exploratory in nature due to the limited subgroup sample size, and the observed associations should therefore be interpreted cautiously until validated in larger independent cohorts. In addition, semen straws were obtained from an AI center, limiting control over donor-level variables, including individual animal variation, semen collection batches, and cryopreservation batch effects [40]. Consequently, although several molecular and phenotypic differences were observed between breeds, these findings cannot be attributed exclusively to breed effects, as potential confounding factors may also have contributed to the observed variation. Future studies should validate these candidate biomarkers in larger independent cohorts, include donor metadata, and evaluate their association with *in vivo* fertility outcomes, such as conception rates following AI, to strengthen their practical relevance. In addition, targeted proteomic validation approaches, including PRM/MRM and Western blotting, are needed to confirm the differential abundance and reliability of the identified candidate markers.

In summary, PE and Saanen frozen–thawed semen differed significantly in viability and membrane integrity, while sperm kinematics were largely comparable. Proteomic profiling suggested breed-associated molecular differences, particularly involving mitochondrial metabolism and fatty acid β-oxidation pathways. Several proteins were identified as putative candidate markers with potential discriminatory ability between breeds, including (ACADVL, ETFB, GPD2, LOC102188264), with an additional structural marker (CYLC1). These findings provide preliminary insight into molecular mechanisms potentially associated with sperm cryotolerance and support the integration of phenotypic and proteomic approaches for future studies on improving semen evaluation and preservation strategies in goats.

## CONCLUSION

PE and Saanen frozen–thawed semen differed significantly in viability and plasma membrane integrity, while sperm kinematic parameters were largely similar. Proteomic profiling revealed breed-associated molecular differences dominated by mitochondrial-related proteins and fatty acid β-oxidation pathways. Candidate biomarkers with discriminative performance (AUC≥0.70) included ACADVL, ETFB, GPD2, LOC102188264, and CYLC1, and several showed significant correlations with semen parameters. These findings highlight mitochondrial metabolism as a key molecular feature associated with post-thaw semen performance and support the potential use of proteomic markers to complement conventional semen evaluation.

## Figures and Tables

**Figure 1:**
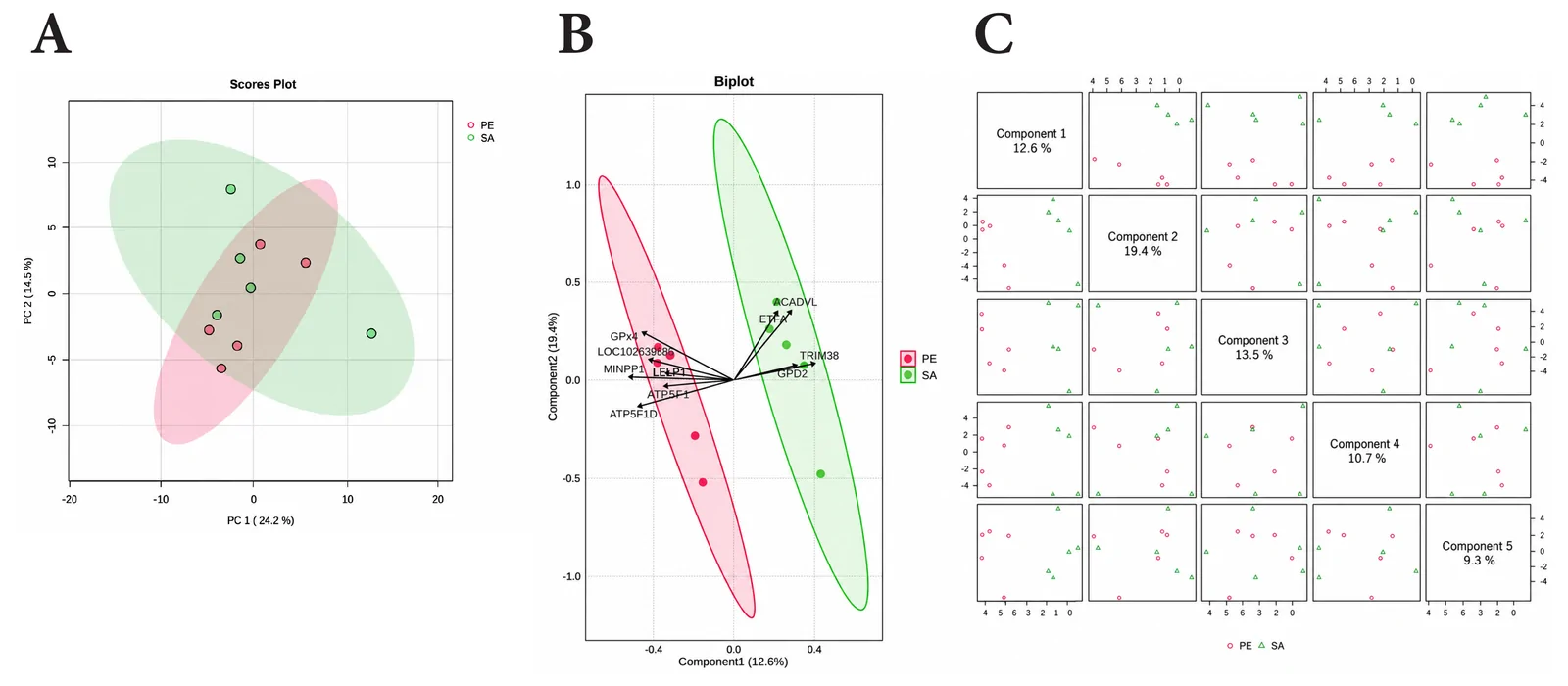
Overview of semen proteomic profiling in Peranakan Etawah (PE) and Saanen goats (SA). (A) PCA score plot showing sample clustering. (B) PLS-DA biplot illustrating group separation and contributing proteins. (C) Permutation test results for PLS-DA model validation. PCA, principal component analysis; PLS-DA, partial least squares–discriminant analysis.

**Figure 2:**
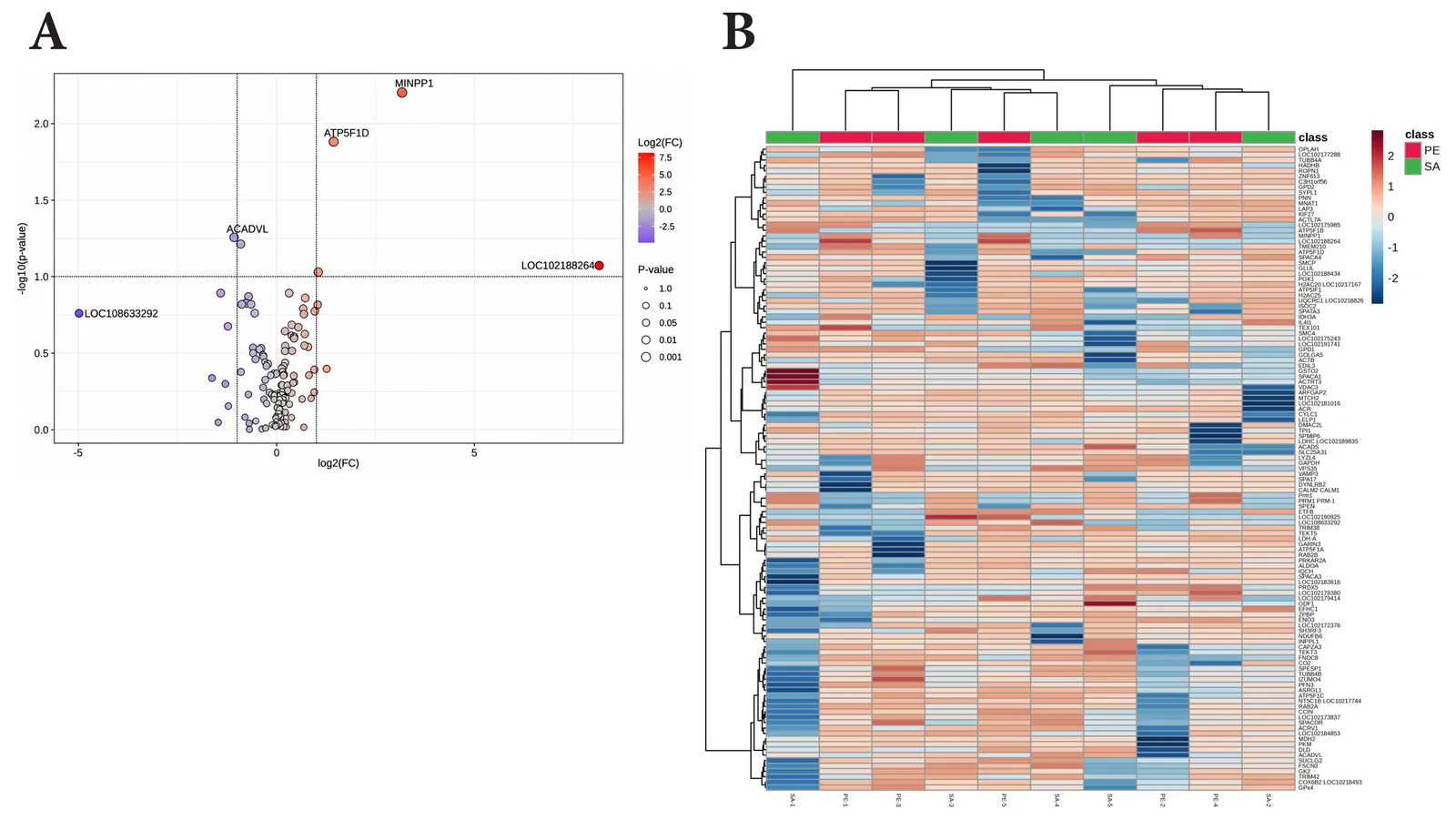
Differential protein abundance between Peranakan Etawah (PE) and Saanen goats (SA). (A) Volcano plot showing significantly altered proteins. (B) Heatmap illustrating hierarchical clustering of differentially abundant proteins.

**Figure 3:**
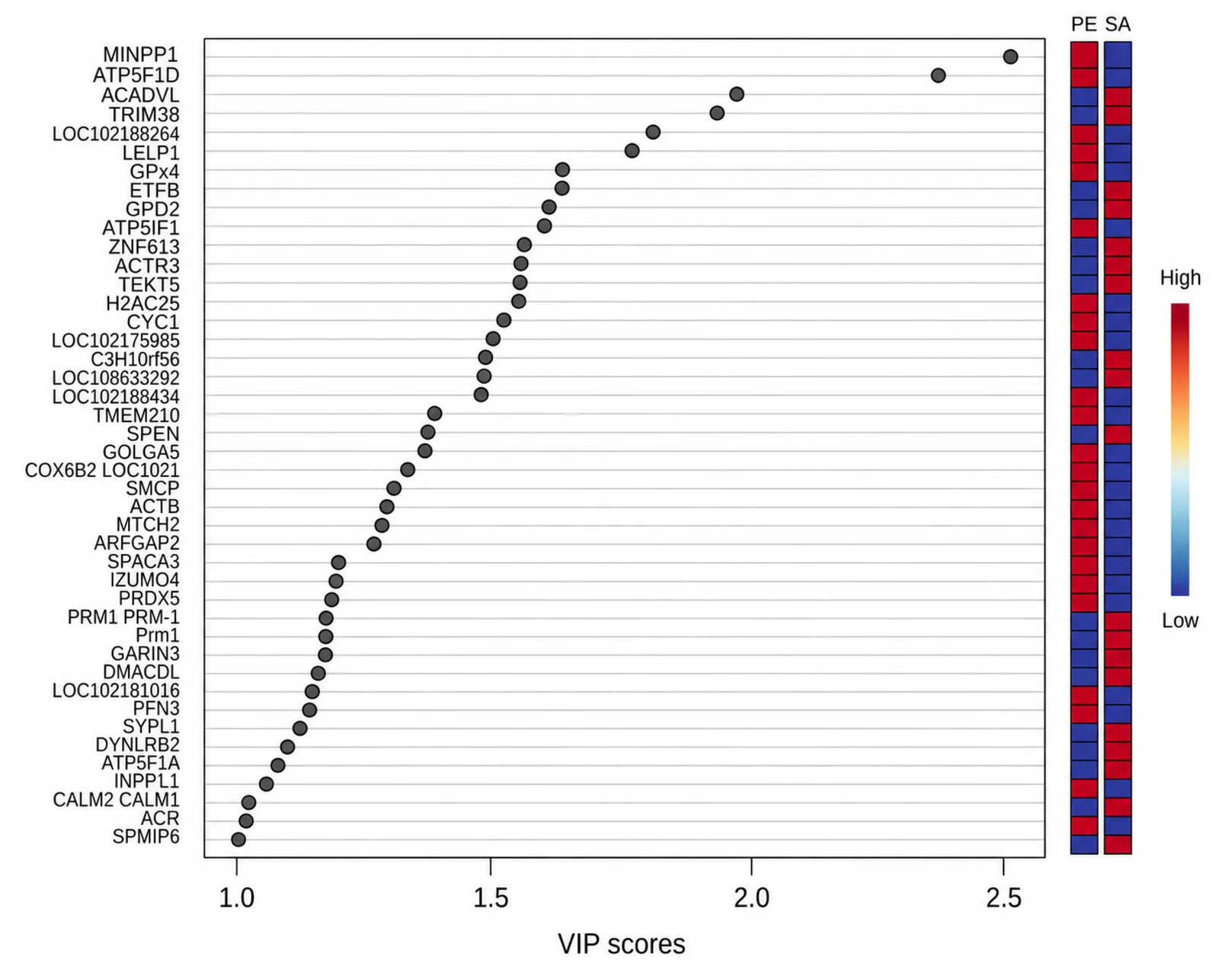
Variable importance in projection (VIP) scores derived from the PLS-DA model showing proteins contributing to discrimination between PE and Saanen frozen–thawed semen samples. Proteins with VIP scores>1 were considered important contributors to group separation. PE, Peranakan Etawah; PLS-DA, partial least squares–discriminant analysis.

**Figure 4:**
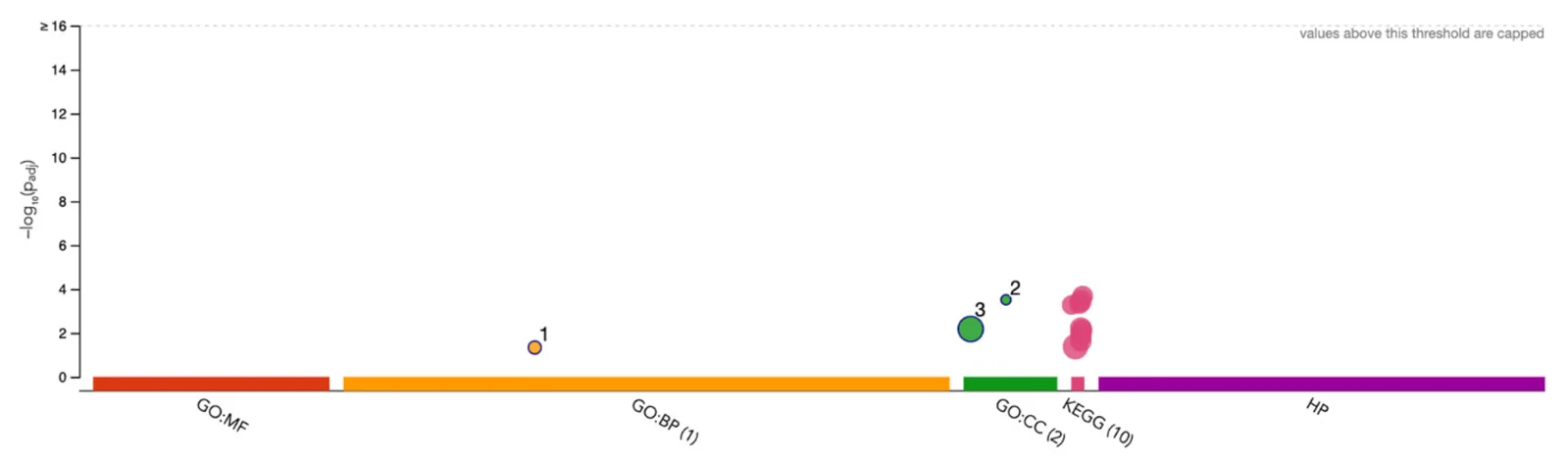
Functional enrichment analysis of differentially abundant proteins identified using g:Profiler. The horizontal axis represents functional annotation categories, and the vertical axis indicates enrichment significance expressed as −log10 adjusted p-values. Each circle represents an enriched term. GO, Gene Ontology; MF, molecular function; BP, biological process; CC, cellular component; KEGG, Kyoto Encyclopedia of Genes and Genomes; HP, Human Phenotype.

**Figure 5:**
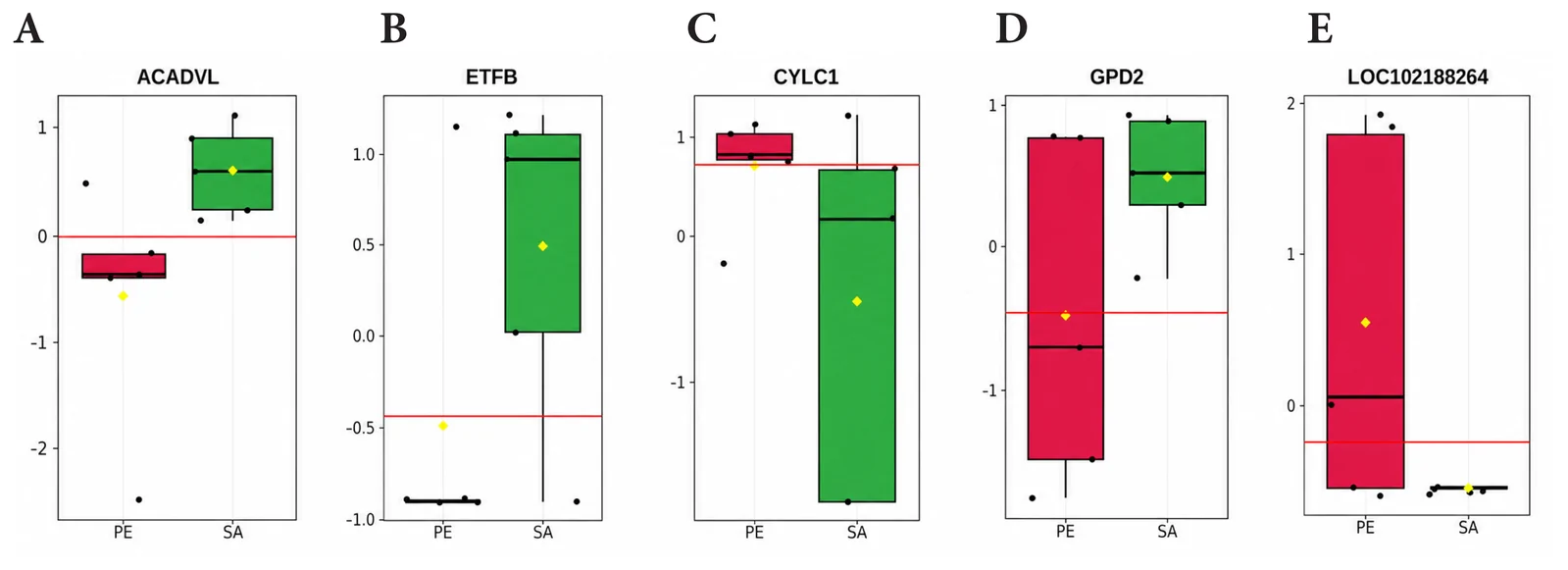
Box plots of the relative abundance of candidate biomarker proteins between Peranakan Etawah (PE) and Saanen goats (SA). (A) ACADVL, (B) ETFB, (C) CYLC1, (D) GPD2, and (E) LOC102188264.

**Figure 6:**
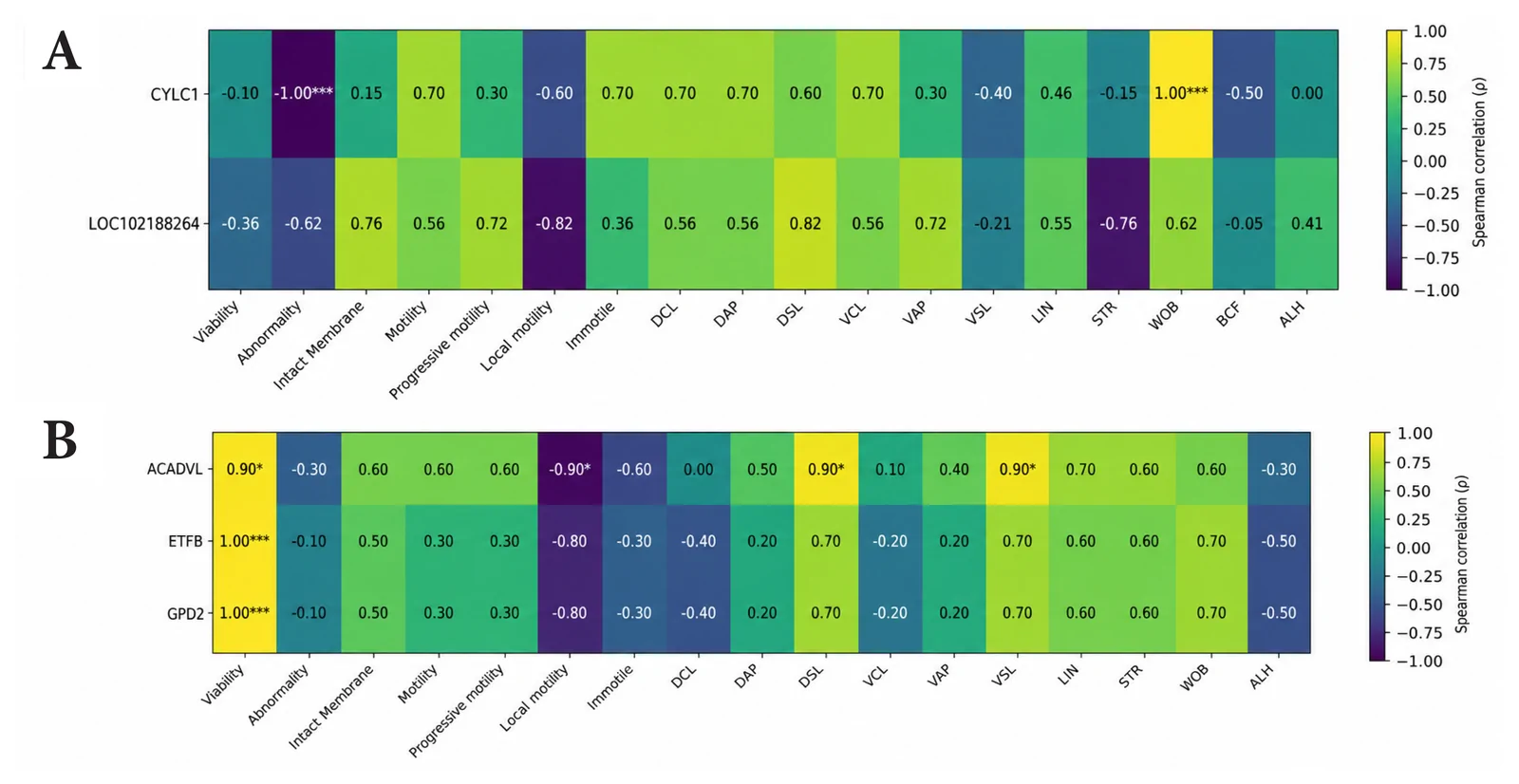
Spearman correlation heatmap between semen parameters and candidate biomarkers in (A) PE and (B) Saanen. * p<0.05; *** p<0.001. CYLC1, Cylicin 1; LOC102188264, Cytochrome b-c1 complex; ACADVL, Acyl-CoA Dehydrogenase Very Long Chain; ETFB, Electron transfer flavoprotein subunit beta; GPD2, Glycerol-3-phosphate dehydrogenase; DCL, distance curved line; DAP, distance average path; DSL, 633 distance straight line; VCL, velocity curved line; VAP, velocity average path; VSL, velocity straight line; 634 LIN, linearity; STR, straightness; WOB, wobble; BCF, beat cross frequency; ALH, amplitude of lateral 635 head displacement; PE, Peranakan Etawah.

**Table 1:** Post-thaw semen quality parameters of frozen semen from Peranakan Etawah (PE) and Saanen goats (SA)

Parameter	PE	SA	p-value
Viability (%)1)	85.60±2.35	81.16±2.77	0.027
Abnormality (%)2)	12.34±0.72	11.05±0.71	0.022
Intact membrane (%)1)	64.23±1.17	55.87±2.52	0.001
Motility (%)1)	76.23±3.64	73.75±3.26	0.290

Data are presented as mean±SD.

Normally distributed variables were analyzed using an independent samples t-test, whereas non-normally distributed variables were analyzed using the Mann–Whitney U test.

1) Independent samples t-test.

2) Mann–Whitney U test.

SD, standard deviation.

**Table 2:** Post-thaw semen kinematic parameters of frozen semen from Peranakan Etawah (PE) and Saanen goats (SA)

Parameter	PE	SA	p-value
Progressive motility (%)1)	62.42±5.60	62.86±5.12	0.900
Local motility (%)2)	14.37±3.59	11.36±2.23	0.157
Immotile (%)2)	23.34±3.53	26.39±3.87	0.228
DCL (μm)1)	40.16±5.60	40.09±5.07	0.985
DAP (μm)1)	24.31±3.07	22.93±2.64	0.468
DSL (μm)1)	16.50±1.32	17.06±2.76	0.696
VCL (μm/s)1)	92.41±12.39	90.39±9.23	0.778
VAP (μm/s)1)	53.79±5.18	52.31±5.02	0.658
VSL (μm/s)1)	35.75±3.91	39.04±5.31	0.301
LIN (%)1)	0.44±0.02	0.43±0.06	0.727
STR (%)1)	0.69±0.05	0.74±0.04	0.135
WOB (%)1)	0.57±0.07	0.58±0.04	0.671
BCF (Hz)1)	23.06±3.94	23.48±1.80	0.838
ALH (μm)1)	5.63±3.27	4.13±0.49	0.367

Data are presented as mean±SD.

Normally distributed variables were analyzed using an independent samples t-test, whereas non-normally distributed variables were analyzed using the Mann–Whitney U test.

1) Mann–Whitney U test.

2) Independent samples t-test.

DCL, distance curved line; DAP, distance average path; DSL, distance straight line; VCL, velocity curved line; VAP, velocity average path; VSL, velocity straight line; LIN, linearity; STR, straightness; WOB, wobble; BCF, beat cross frequency; ALH, amplitude of lateral head displacement; SD, standard deviation.

**Table 3:** Cross-validation performance of the PLS-DA model for Peranakan Etawah and Saanen frozen-thawed semen proteomic profiles

Measure	1 Component	2 Components	3 Components	4 Components	5 Components
Accuracy	0.40	0.40	0.40	0.40	0.40
R^2^	0.90389	0.98758	0.99918	0.99999	1.00000
Q^2^	−0.55699	−0.44877	−0.43939	−0.44132	−0.42230

PLS-DA, partial least squares–discriminant analysis.

**Table 4:** Functional enrichment analysis of differentially abundant proteins in Peranakan Etawah and Saanen goats

Term ID	Term name	Padj	Protein
GO:0033539	Fatty acid beta-oxidation using acyl-CoA dehydrogenase	4.619E-02	ACADVL; ETFB
GO:0043159	Acrosomal matrix	3.090E-04	CYLC1
GO:0005739	Mitochondrion	6.611E-03	ACADVL; LOC102188264; ETFB; GPD2; ATP5IF1

GO, Gene Ontology.

**Table 5:** Candidate biomarkers between Peranakan Etawah (PE) and Saanen goats (SA)

Gene	Accession	Protein	Group	VIP	Pathway	AUC
*ACADVL*	A0A452G2G4	Acyl-CoA Dehydrogenase Very Long Chain	SA	1.97651	GO:0033539; GO:0005739	0.92
*ETFB*	A0A8C2QZW0	Electron transfer flavoprotein subunit beta	SA	1.63491	GO:0033539; GO:0005739	0.76
*CYLC1*	A0A452ESC9	Cylicin 1	PE	1.52117	GO:0043159	0.72
*GPD2*	A0A452F334	Glycerol-3-phosphate dehydrogenase	SA	1.6103	GO:0005739	0.76
*ATP5IF1*	A0A452DUN3	ATPase inhibitor, mitochondrial	PE	1.59993	GO:0005739	0.68
*LOC102188264*	A0A8C2S1J5	Cytochrome b-c1 complex subunit 1, mitochondrial	PE	1.81216	GO:0005739	0.8

VIP, variable importance in projection; AUC, area under the curve; GO, Gene Ontology.

## Data Availability

Upon reasonable request, the datasets of this study can be available from the corresponding author.
